# Comparison between plate cage system and stand-alone cage in the treatment of cervical spondylotic myelopathy patients with cervical kyphosis: clinical and radiographic outcomes

**DOI:** 10.3389/fsurg.2026.1749161

**Published:** 2026-02-18

**Authors:** Chao Wang, Shu Liu, Jiabin Yuan, Zhicai Shi

**Affiliations:** 1 Department of Orthopedics, The Shanghai Tenth People’s Hospital of Tongji University, Shanghai, China; 2 Department of Orthopedics, Changhai Hospital, Naval Medical University, Shanghai, China

**Keywords:** anterior cervical discectomy and fusion, cervical kyphosis, plate cage system, sagittal alignment, stand-alone cage

## Abstract

**Background:**

To compare mid-term outcomes of plate cage systems (PC) vs. stand-alone cages (SA) in two-level ACDF for cervical spondylotic myelopathy (CSM) with cervical kyphosis, and to assess the influence of preoperative segmental kyphosis reducibility on sagittal alignment maintenance.

**Methods:**

This retrospective cohort analyzed 130 patients (SA = 64, PC = 66) with ≥24-month follow-up. Radiographic parameters (cervical lordosis [CL], fusion segmental lordosis [FSL], disc wedge, C2 SVA) and clinical outcomes (NDI, VAS, JOA, EQ-5D, dysphagia) were assessed preoperatively, postoperatively, and at final follow-up. Patients were stratified based on preoperative reducibility of segmental kyphosis.

**Results:**

Both groups showed comparable clinical improvement (*p* < 0.05) and fusion rates (93.8% SA vs. 95.4% PC, *p* = 0.667). SA had shorter operation time (78.0 ± 11.5 vs. 86.6 ± 12.5 min, *p* < 0.001), less blood loss (193.0 ± 85.6 vs. 256.7 ± 110.3 mL, *p* < 0.001), and lower dysphagia incidence (12.5% vs. 20.3%, *p* = 0.014). However, the PC group demonstrated superior maintenance of cervical sagittal alignment in CL (13.6° ± 0.9° vs. 12.3° ± 2.4°, *p* < 0.001), FSL (13.2° ± 1.2° vs. 11.9° ± 2.9°, *p* = 0.001), and disc wedge (10.2° ± 1.3° vs. 8.9° ± 2.6°, *p* < 0.001) at follow-up, particularly in patients with non-reducible kyphosis (*p* < 0.05).

**Conclusion:**

SA offers operative efficiency advantages, while PC is more effective in maintaining sagittal alignment, especially in rigid kyphosis. PC is recommended for patients with non-reducible cervical kyphosis.

## Introduction

Cervical spondylotic myelopathy (CSM), generally caused by compression of the cervical spinal cord, represents a collection of pathological entities and results in a clinical syndrome including gait imbalance, loss of hand dexterity, and/or sphincter dysfunction (1). Because of a shorter hospital stay and more successful restoration of cervical lordosis than posterior surgery, anterior cervical discectomy and fusion (ACDF) is considered as the gold standard for patients with ineffective conservative treatment or obvious neurological symptoms (2, 3). Both stand-alone cage (SA) and plate cage system (PC) have demonstrated improvement in neurological function and health-related outcomes (3–5). Previous studies have shown the advantage of stand-alone cage in decreased operation time and lower incidence of postoperative dysphagia (3, 4, 6). Controversy, however, remains regarding the best method for ACDF considering that the plate cage system can effectively prevent the cage subsidence, and maintain normative sagittal alignment of cervical spine (3, 6, 7).

Sagittal alignment balance, especially kyphotic deformity, should always be considered during surgical treatment of CSM patients to decrease cord tension induced by kyphosis (8). Kato et al. pointed out that CSM patients with cervical kyphosis postoperatively exhibit significantly lower Short Form-36 Health Survey (SF-36) physical component score (9). Although the superiority of PC in maintaining sagittal alignment has been documented (3, 7, 10), the clinical and radiological mid-term outcomes of the two methods, to our knowledge, applied to CSM patients with cervical kyphosis are still unclear.

The current study aims to evaluate and contrast the clinical and radiological outcomes observed in CSM patients with cervical kyphosis who underwent ACDF with either a stand-alone cage or a plate cage system. Furthermore, fused segment lordosis can further be classified into the reducible or non-reducible group depending on the ability of recovering lordosis in preoperative extension image (11). So, we endeavor to explore the potential use of preoperative fused segment lordosis classification, specifically whether it is reducible or non-reducible, as a predictive factor for the reconstruction of sagittal cervical alignment during the surgical procedure.

## Materials and methods

### Patient population

This retrospective study was approved by the Institutional Committee for Clinical Studies. We retrospectively reviewed CSM patients with cervical kyphosis at our institution from May 2019 to April 2021. A total of 130 patients (63 men, 67 women) underwent two-level ACDF with either stand-alone cages (SA group, *n* = 64) or plate-cage systems (PC group, *n* = 66). Group allocation was based on surgeon preference and implant availability after ≥3 months of failed conservative treatment. To better appreciate the effects, we selected only patients who had undergone fusion surgery of two segments which included the apex of kyphosis between C3-6. Inclusion and exclusion criteria are listed in Table 1. Sixty-four patients underwent ACDF with stand-alone spacer (SA group) and 66 with plate cage system (PC group). Patient demographics at baseline are presented in Table 2. We quantified bone mineral density via dual-energy x-ray absorptiometry (DXA), with osteoporosis diagnosed when the lowest T-score among lumbar spine (L1-4), femoral neck, and proximal femur measurements reached ≤ −2.5 standard deviations (12).

**Table 1:** Inclusion and exclusion criteria.

Inclusion criteria	Exclusion criteria
Age between 45 and 75 years	Previous cervical spine surgery
Diagnosis of cervical spondylotic myelopathy (CSM) with concomitant cervical kyphosis	More than two cervical levels requiring treatment
refractory to at least 3 months of conservative treatment	Presence of cervical lordosis (C2–7 Cobb angle ≥0°)
Correlating findings on magnetic resonance imaging (MRI) on two cervical levels, including the apex of kyphosis;	Diagnosis of cervical spondylotic radiculopathy (CSR)
Eligible for both treatments	Congenital cervical anomalies (e.g., segmentation failure, hemivertebrae)
	Concurrent cervical trauma or neoplastic lesions
	Rheumatoid arthritis, known malignancy, active infection, or other systemic disease

**Table 2:** Demographics at baseline.

Patient characteristics	SA, *n* = 64	PC, *n* = 66	*P*
Sex (M/F)	31/33	34/32	0.859
Age, mean (SD)	63.4 (5.9)	63.7 (4.8)	0.733
Body mass index (BMI), mean (SD)	24.4 (2.8)	25.3 (3.4)	0.136
Bone mineral density (DXA, T scores), mean (SD)	−1.86 (0.81)	−2.07 (0.81)	0.127
Symptom duration, *n* (%)			0.130
<6 mo	29 (45.3)	31 (47.0)	
6 to 12 mo	28 (43.8)	27 (40.9)	
>1 y	7 (10.9)	8 (12.1)	
Analgesic medication, *n* (%)			0.059
Regularly	28 (43.8)	30 (45.5)	
Irregularly	30 (46.9)	29 (43.9)	
No analgesics	6 (9.3)	7 (10.6)	

SD, standard deviation.

### Operative procedure

All procedures were performed by a senior spinal surgeon with over 15 years of experience in spine surgery. Under general anesthesia, patients underwent a standardized anterior cervical discectomy and fusion (ACDF) via the Smith-Robinson approach. A right-sided anterolateral incision was utilized to expose the pathological disc levels. Following meticulous localization using intraoperative fluoroscopy, complete discectomy and neurological decompression were achieved. Cartilaginous endplates were meticulously decorticated using a high-speed pneumatic drill, while subchondral bone integrity was preserved to prevent graft subsidence. Osteophyte resection and posterior longitudinal ligament release were selectively performed when required for adequate cord decompression.

In the SA group, stand-alone cages with integrated screw fixation (Zero-P, DePuy Synthes) were implanted, whereas the PC group received polyetheretherketone (PEEK) interbody cages (Skyline, DePuy Synthes) combined with anterior cervical plating systems (Bengal, DePuy Synthes). All cages were pre-packed with allograft bone grafting material prior to insertion. Full ambulation was initiated on postoperative day 2, with external immobilization maintained using a rigid cervical collar for 4–6 weeks.

### Clinical outcome

Operation time, blood loss, complications and length of hospital stay were collected perioperatively. The degree of dysphagia was evaluated by Bazaz et al. grading system (13) at 48 h postoperatively and at the 3-, 6-month follow-up. The Neck Disability Index (NDI) score (ranging from 0 to 50) was used as a primary outcome variable (14) and health-related outcomes were evaluated using the Visual Analogue Scale (VAS) for arm pain (VAS-arm) and for neck pain (VAS-neck), the Japanese Orthopedic Association (JOA) score (ranging from 1 to 17), and the EuroQol Five-Dimension Scale (EQ-5D). Assessments were conducted preoperatively, as well as 3 month, 6 months, 1 year, and 2 years postoperatively. The recovery rate (RR) of the JOA score was calculated by the following formula: [RR = (postoperative−preoperative JOA score)/(17−preoperative JOA score) × 100%] (15).

### Radiographic assessment

Cervical kyphosis was defined as cervical lordosis (C2-7 Cobb) <0° in C-spine lateral neutral x-ray and was not the compensation of global spine imbalance confirmed by whole spine x-ray (8, 11, 16). In this study, radiographs were taken preoperatively and during each follow-up session. Specifically, plain lateral radiographs in neutral, flexion, and extension positions were obtained for comprehensive assessment. Fusion rate, cage subsidence, and ASD were systematically assessed at the 2-year postoperative follow-up. On dynamic radiographs, the radiographic fusion criteria consisted of interspinous motion (ISM) < 1 mm and superjacent ISM ≥4 mm (17). Cage Subsidence was generally defined as ≥ 3 mm loss of height comparing the direct postoperative intervertebral height with the intervertebral height at the last follow-up moment (18, 19). Adjacent segment disease (ASD) was characterized by degenerative changes observable on x-rays, encompassing: (1) formation or increased anterior osteophytes; (2) new or increased disc space narrowing (>30%); (3) new or increased anterior longitudinal ligament calcification; (4) growth of radial osteophytes (20).

**Figure 1:**
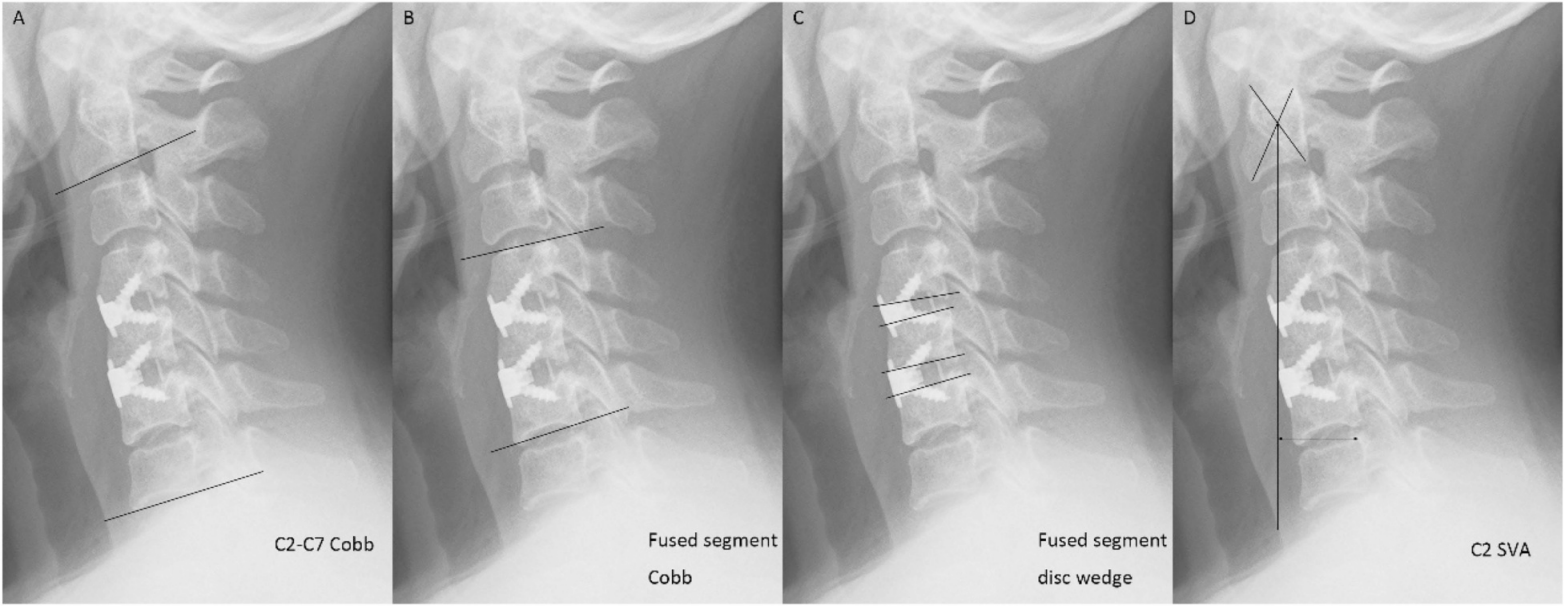
Illustration showing the method of cervical sagittal alignment measurements. **(A)** C2-C7Cobb angle; **(B)** fused segment Cobb angle (in this case, C4-C6); **(C)** fused segment disc wedge (in this case, C4/5 + C5/6); **(D)** C2-C7 sagittal vertical axis.

Lateral x-ray films were used to measure the sagittal alignment of the cervical spine. Cervical lordosis (C2-C7, CL) and fusion segmental lordosis (FSL) were measured in the lateral radiograph of neutral position (Cobb method). The fused segment disc wedge was calculated by adding up the Cobb angles of the three operated discs. C2 sagittal vertical axis (C2 SVA), which can be defined regionally using the distance between a plumb line dropped from the centroid of C2 and the posterosuperior aspect of C7, was obtained at the same time (Figure 1). Those radiographic metrics were repeated 3 times, and the results were averaged. To achieve blinding, identifying information on radiographs was removed, and they were randomly assigned to examiners, each with at least 3 years of clinical experience in spinal surgery.

### Statistical analysis

The sample size was calculated with the Neck Disability Index (NDI) as the primary outcome variable in a superiority design. The standard deviation (SD) of NDI was set at 18, determined from our institutional data and relevant literature (21, 22). The minimal detectable difference (effect size) was set at 10. With a significance level of 0.05 and a power of 80%, the initial calculation indicated that 51 patients were needed per group. However, considering potential loss of follow - up, possible noncompliance of patients, and the complexity of surgical procedures that might introduce additional variability, we adjusted the sample size. In this study, a total of 130 patients were included, which is expected to provide sufficient statistical power to accurately assess the differences in NDI scores between the plate cage and stand—alone cage groups during anterior cervical discectomy and fusion (ACDF) for the treatment of cervical spondylosis.

To ensure the reliability and validity of the study results, a comprehensive statistical analysis was conducted on the data obtained from both groups. Descriptive statistics were used to analyze data in both groups. For group - to - group comparisons, an independent *t* test was applied to continuous variables meeting normality and variance—homogeneity assumptions, the chi—square test was used for categorical variables, and the Mann - Whitney U test or Fisher exact test was chosen when parametric test assumptions were violated.

To analyze ordinal data within groups, the Wilcoxon test and Friedman analysis of variance were employed. For between—group repeated—measurement data, the Kruskal - Wallis analysis of variance was used. A *p*-value < 0.05 was regarded as statistically significant. In subgroup analyses, the Bonferroni correction was used to adjust the significance level. SPSS 22.0 software (IBM, Armonk, New York, USA) was used for statistical analysis.

## Results

### Surgical outcomes

Operative data are summarized in Table 3. While no significant difference was observed in the length of hospital stay or surgical level between the SA and PC groups, the SA group demonstrated significantly shorter operative time (78.0 ± 11.5 vs. 86.5 ± 12.5 min, *p* < 0.001) and reduced intraoperative blood loss (193.0 ± 85.6 vs. 256.7 ± 110.3 mL, *p* < 0.001) compared to the PC group.

**Table 3:** Patient perioperative parameters.

Variable	SA	PC	*P*
Operation time (min), mean (SD)	78.0 (11.5)	86.5 (12.5)	<0.001*
Blood loss (mL), mean (SD)	193.0 (85.6)	256.7 (110.3)	<0.001*
Surgical level, *n* (%)			0.483
C4/5	34 (53.1)	31 (47.0)	
C5/6	30 (46.9)	35 (53.0)	
Lenth of hospital stays (d), mean (SD)	7.3 (2.3)	7.7 (1.9)	0.333

* Significantly different between the SA and the PC group.

The complications of these patients are summarized in Table 4. In the SA group, postoperative complications included dysphagia (8 cases), subcutaneous hematoma (1 case), wound infection (1 cases) and hoarseness (3 case). In the PC group, postoperative complications included dysphagia (20 cases), cerebral fluid leakage (1 case), epidural hematoma (1 cases), subcutaneous hematoma (2 case), wound infection (1 cases), Horner syndrome (1 case) and hoarseness (3 cases). There was no implant failure or new neurological deficit in both groups.

**Table 4:** Adverse events and complications.

Complication	SA	PC
Dysphagia, *n* (%)*		
48-h	8 (12.5)	20 (30.3)
3-mo	6 (9.4)	15 (22.7)
6-mo	1 (0.02)	8 (12.1)
Cerebral fluid leakage, *n* (%)	0	1 (1.5)
Epidural hematoma, *n* (%)	0	1 (1.5)
Subcutaneous hematoma, *n* (%)	1 (1.6)	2 (3.0)
Wound infection, *n* (%)	1 (1.6)	1 (1.5)
Horner syndrome, *n* (%)	0	1 (1.5)
Hoarseness, *n* (%)	3 (4.7)	3 (4.5)

* Significantly different between the SA and the PC group (*p* < 0.05).

The incidence of most postoperative complications did not differ significantly between the two groups. However, the PC group demonstrated a statistically higher rate of dysphagia compared to the SA group (*p* = 0.014). There were 8 patients developed mild dysphagia in the SA group and 20 patients developed dysphagia (12 mild, 8 moderate) in the PC group 48 h after surgery. At 3-month follow-up, 6 patients in the SA group showed mild dysphagia and only 1 at 6-month follow-up, however 15 patients showed dysphagia (9 mild, 6 moderate) in the PC group at 3-month follow-up, and the mild symptom still present in 8 patients at 6-month follow-up. Overall, the PC group exhibited a significantly higher incidence of postoperative dysphagia than the SA group throughout the follow-up period.

### Clinical outcomes

**Figure 2:**
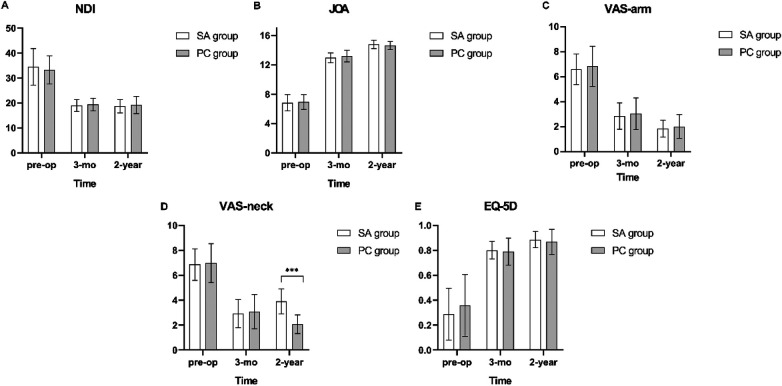
**(A–E)** diagrams of outcome in NDI, JOA, VAS-arm, VAS-neck and EQ-5D. Columns represent medians, and whiskers show SD preoperatively, after 3 months and 2 years, respectively; NDI, Neck Disability Index; JOA, Japanese Orthopaedic Association score; VAS, visual analog scale; EQ-5D, EuroQol Five Dimension Scale. ***p < 0.01.

There were no significant differences between 2 groups preoperatively in NDI, VAS-arm, JOA, or EQ-5D score (*p* > 0.05). All outcome measures improved after surgery, regardless of the treatment strategy (Table 5). The NDI decreased significantly from 18.2 ± 1.8 points at baseline to 9.4 ± 0.8 and 9.3 ± 1.1 points 3-month and 2-year postoperative, respectively, in the SA group, from 17.8 ± 1.4 points to 9.7 ± 0.9 and 9.6 ± 1.2 points in the PC group, and there was no significant difference in the absolute value of NDI, nor in the decline of NDI among theme (Figure 2A). Likewise, secondary parameters, including JOA, VAS-arm, EQ-5D, also significantly improved after surgery and showed a comparable result in both groups (Figures 2B–D). At the 2-year follow-up, the SA group showed a more pronounced VAS-neck compared with the PC group (3.9 ± 1.0 and 2.1 ± 0.8, respectively), which, however, failed reach the minimal clinically important difference (MCID) of 2.6 points on the 10-point VAS scale (23) (Figure 2E).

**Table 5:** Patient clinical outcomes.

Variable	SA	PC	*P*
NDI scores, mean (SD)			
Preoperative	34.5 (7.3)	33.2 (5.6)	0.261
Postoperative 3 months	18.9 (2.4)**	19.3 (2.5)**	0.358
Postoperative 2 years	18.7 (2.7)**	19.2 (3.4)**	0.395
JOA scores, mean (SD)			
Preoperative	6.8 (1.1)	6.9 (1.0)	0.606
Postoperative 3 months	13.0 (0.7)**	13.2 (0.8)**	0.073
Postoperative 2 years	14.8 (0.6)**^,^***	14.6 (0.5)**^,^***	0.107
VAS-arm scores, mean (SD)			
Preoperative	6.6 (1.2)	6.9 (1.6)	0.346
Postoperative 3 months	2.9 (1.1)**	3.1 (1.3)**	0.365
Postoperative 2 years	1.9 (0.7)**^,^***	2.0 (1.0)**^,^***	0.283
VAS-neck scores, mean (SD)			
Preoperative	6.9 (1.3)	7.0 (1.6)	0.616
Postoperative 3 months	2.9 (1.1)**	3.1 (1.4)**	0.445
Postoperative 2 years	3.9 (1.0)**^,^***	2.1 (0.8)**^,^***	<0.001*
EQ-5D scores, mean (SD)			
Preoperative	0.29 (0.21)	0.36 (0.25)	0.088
Postoperative 3 months	0.80 (0.07)**	0.79 (0.11)**	0.543
Postoperative 2 years	0.89 (0.07)**^,^***	0.87 (0.10)**^,^***	0.257

VAS, indicates visual analogue scale, JOA score indicates Japanese Orthopaedic Association score for cervical myelopathy, NDI means neck disability index, EQ-5D indicates EuroQol Five Dimension Scale.

* Significantly different between the SA and the PC group.

** Significantly different from preoperative baseline (*p* < 0.05).

*** Significantly different from postoperative 3 months (*p* < 0.05).

### Radiographic outcomes

Radiographic outcomes are detailed in Table 6. At the 2-year follow-up, fusion rates were comparable between the SA and PC groups (93.8% [60/64] vs. 95.4% [63/66], *p* = 0.667). Adjacent segment disease (ASD) occurred in 8 patients (12.3%) in the SA group and 7 patients (10.6%) in the PC group (*p* = 0.735), though none required revision surgery. Furthermore, cage subsidence was more frequently observed in the SA group than in the PC group (14.1% [9/64] vs. 9.1% [6/66]); however, no statistically significant intergroup difference was observed (*p* = 0.289).

**Table 6:** Patient radiologic outcomes.

Variable	SA	PC	*P*
Fusion rate (%)	93.8 (60/64)	95.4 (63/66)	0.667
ASD (%)	12.3 (8/64)	10.6 (7/66)	0.735
Cage subsidence (%)	14.1 (9/64)	9.1 (6/66)	0.375
C2-C7 Cobb angle (°), mean (SD)			
Preoperative	−6.2 (0.8)	−6.3 (1.2)	0.364
Postoperative 3 months	14.9 (0.7)**	14.9 (0.8)**	0.871
Postoperative 2 years	12.3 (2.4)**^,^***	13.6 (0.9)**^,^***	<0.001*
Fused segment Cobb (°), mean (SD)			
Preoperative	−8.0 (0.8)	−8.1 (1.0)	0.528
Postoperative 3 months	13.1 (0.6)**	13.2 (0.8)**	0.309
Postoperative 2 years	11.9 (2.9)**^,^***	13.2 (1.2)**	0.001*
Fused segment disc wedge (°), mean (SD)			
Preoperative	−7.5 (2.1)	−8.0 (1.2)	0.130
Postoperative 3 months	12.0 (0.7)**	12.1 (1.1)**	0.328
Postoperative 2 years	8.9 (2.6)**^,^***	10.2 (1.3)**^,^***	<0.001*
C2-C7 SVA (mm), mean (SD)			
Preoperative	18.7 (1.1)	18.8 (1.9)	0.593
Postoperative 3 months	19.8 (1.0)**	20.2 (1.9)**	0.127
Postoperative 2 years	24.9 (1.3)**^,^***	25.3 (2.4)**^,^***	0.233

SVA, sagittal vertical axis.

* Significantly different between the SA and the PC group.

** Significantly different from preoperative baseline (*p* < 0.05).

*** Significantly different from postoperative 3 months (*p* < 0.05).

Preoperative cervical sagittal alignment parameters—including CL, FSL, fused segment disc wedge, and C2 SVA—showed no significant differences between the SA and PC groups (*p* = 0.364, 0.528, 0.130, and 0.593, respectively; Table 6). Postoperatively, both groups achieved significant correction of sagittal alignment, with comparable improvements between cohorts (*p* > 0.05). However, at final follow-up, the PC group maintained superior cervical alignment: CL (13.6° ± 0.9° vs. 12.3° ± 2.4°; *p* < 0.001), FSL (13.2° ± 1.2° vs. 11.9° ± 2.9°; *p* = 0.001), and fused segment disc wedge (10.2 ± 1.3° vs. 8.9° ± 2.6°; *p* < 0.001). These findings demonstrate significantly greater preservation of sagittal correction in the PC group. No intergroup difference in postoperative C2 SVA was observed (*p* = 0.233).

A comparative analysis of radiographic outcomes between patients with osteoporosis (OP) and non-osteoporotic (non-OP) individuals revealed notable disparities. Non-OP patients exhibited a marked improvement in FSL, progressing from a baseline mean of −8.0° ± 0.9° to 12.8° ± 2.0° at the 2-year follow-up. In contrast, OP patients showed less pronounced gains, with FSL increasing from −8.3° ± 0.9° to 12.0° −8.3° ± 2.7°. Statistically significant difference in FSL (*p* = 0.044) was observed between OP and non-OP cohorts at final follow-up, underscoring the adverse impact of osteoporosis on postoperative alignment restoration.

Furthermore, preoperative stratification based on reducibility of fused segment lordosis yielded distinct postoperative outcomes (Table 7). In patients with non-reducible kyphosis (defined by persistent segmental malalignment on extension radiographs), the SA group demonstrated significantly lower cervical lordosis (CL), fusion segmental lordosis (FSL), and fused segment disc wedge compared to the PC group (*p* < 0.05 for all parameters). Conversely, in reducible cases (segmental lordosis restored on preoperative extension imaging), no significant differences in cervical sagittal alignment were observed between SA and PC groups (*p* > 0.05). These results highlight the critical influence of preoperative reducibility on the efficacy of SA in maintaining corrective alignment, particularly in biomechanically challenging non-reducible deformities.

**Table 7:** Comparison of sagittal alignment by subgroups.

Subgroups	Variables, mean (SD)	SA	PC	*P*
Reducible	n	53/64	53/66	
	C2-C7 Cobb angle (°)			
	Preoperative	−6.2 (0.8)	−6.5 (1.2)	0.139
	Postoperative 3 months	14.9 (0.8)**	15.0 (0.8)**	0.442
	Postoperative 2 years	13.4 (0.9)**^,^***	13.6 (0.9)**^,^***	0.123
	Fused segment Cobb (°)			
	Preoperative	−8.0 (0.8)	−8.3 (1.1)	0.223
	Postoperative 3 months	13.1 (0.7)**	13.4 (0.8)**	0.124
	Postoperative 2 years	13.2 (0.7)**^,^***	13.3 (1.3)**^,^***	0.367
	Fused segment disc wedge (°)			
	Preoperative	−7.8 (0.8)	−8.2 (1.2)	0.068
	Postoperative 3 months	12.0 (0.8)**	12.3 (1.1)**	0.116
	Postoperative 2 years	10.0 (0.8)**^,^***	10.4 (1.4)**^,^***	0.073
Non-reducible	*N*	11/64	13/64	
	C2-C7 Cobb angle (°)			
	Preoperative	−6.3 (0.7)	−5.8 (0.9)	0.238
	Postoperative 3 months	14.9 (0.8)**	14.5 (0.6)**	0.197
	Postoperative 2 years	7.4 (0.8)**^,^***	13.2 (0.5)**^,^***	<0.001*
	Fused segment Cobb (°)			
	Preoperative	−8.0 (0.7)	−8.0 (0.6)	0.135
	Postoperative 3 months	13.0 (0.5)^△^	12.7 (0.6)^△^	0.350
	Postoperative 2 years	5.9 (0.6)**^,^***	12.5 (0.7)**^,^***	<0.001*
	Fused segment disc wedge (°)			
	Preoperative	−6.2 (1.8)	−7.3 (0.7)	0.446
	Postoperative 3 months	11.9 (0.6)**	11.5 (0.5)**	0.102
	Postoperative 2 years	3.4 (0.7)**^,^***	9.4 (0.7)**^,^***	<0.001*

* Significantly different between the SA and the PC group.

** Significantly different from preoperative baseline (*p* < 0.05).

*** Significantly different from postoperative 3 months (*p* < 0.05).

## Discussion

Anterior cervical discectomy and fusion (ACDF) is an established surgical treatment for cervical spondylotic myelopathy (24, 25), with SA and PC being the two most commonly used reconstruction strategies. With the increasing prevalence of cervical kyphosis in the modern population, CSM patients presenting with concomitant sagittal malalignment have become increasingly common, yet the optimal construct selection for this subgroup remains unclear.

In the present study, both the SA and the PC groups showed similarly short length of hospital stay, which was a reflection of rapid recovery due to minimal muscular dissection (2). However, in agreement with published reports (25, 26), the SA group showed a significantly less operation time and blood loss partially due to a smaller incision, minor disruption and no plate placed.In contrast, PC demonstrated superior mid-term maintenance of cervical sagittal alignment, particularly in patients with non-reducible kyphotic deformities, providing clinically relevant insights for construct selection in this challenging population.

Achieving solid interbody fusion stands as a critical objective in ACDF, as failure to establish robust fusion may compromise clinical outcomes (27). A stable fusion construct preserves cervical spine stability, mitigates pseudoarthrosis-related neck pain, and prevents intervertebral height loss. Furthermore, maintaining adequate disc space height reduces the risk of secondary foraminal stenosis, thereby alleviating nerve root compression. A systematic review by Zhang et al. (24) involving nine high-quality studies (580 patients) reported fusion rates exceeding 90% for both SA and PC constructs, consistent with our findings of 91.3% (SA) and 95.2% (PC) (*p* = 0.605). Importantly, these high fusion rates correlated with significant improvements in clinical outcomes, including NDI, JOA scores, VAS-arm and EQ-5D, at all follow-up intervals (*p* < 0.001). No statistically significant differences in these parameters were observed between the two groups, which aligns with the findings of numerous previous studies (28, 29). In contrast, Zavras et al. (30) conducted a prospective randomized trial comparing PC and SA constructs in 42 ACDF patients, demonstrating superior early outcomes (NDI and VAS-neck scores at 6 months) in the PC group, likely attributable to enhanced stability from anterior plating. Similarly, our study revealed higher VAS-neck scores in the SA group compared to the PC group at final follow-up (3.4 ± 1.0 vs. 2.6 ± 0.9, *p* = 0.001). Although the higher VAS-neck scores observed in the SA cage group at final follow-up did not exceed the established minimal clinically important difference (MCID) threshold of 2.5 points for VAS-neck (23), this finding may still reflect subtle differences in biomechanical stability and segmental load sharing between constructs. Such differences may represent an early signal of divergent long-term trends rather than immediate clinically meaningful deterioration.

Consensus has been reached that the incidence of dysphagia after surgery in the PCC group is substantially higher than that in the SA group, and our findings were consistent with previous reports. The incidence of dysphagia varies from 2% to 83% according to the method of assessment (31), and lower dysphagia rate has been emphasized by many reports including this study when the stand-alone cages were used (32). The stimulation of throat by plate, as well as the longer operation time and traction, are the main reasons for the high incidence of dysphagia (3, 7). The severe degree of dysphagia-related symptoms, based on the Bazaz et al. grading system are categorized as none (no episodes of swallowing problems), mild (rare episodes of dysphagia), moderate (occasional swallowing difficulty with specific food), and severe (frequent difficult swallowing with majority of food) (13). Using the EAT-10 score and Hyodo-Komagane score with endoscopic evaluation, a recent article pointed out that aging and smoking are significant risk factors for transient dysphagia, while preoperative local kyphosis angles of C3–C4 and C4–C5 and change in the kyphotic angle at C4/C5 during surgery may be a key alignment of risk factors for postoperative persistent dysphagia (31). As a systematic review of prospective randomized controlled trials noted, perioperative intravenous and local steroid use can reduce the incidence and severity of early dysphagia after anterior cervical spine surgery, especially for multilevel surgeries (33). All patients with dysphagia in this study recovered with conservative management, including intravenous steroid application and restriction of esophageal irritant food within 3 months.

Cervical lordosis plays a critical role in maintaining sagittal balance between the head and spine (34). Loss of physiological lordosis or progression to kyphosis may trigger paraspinal muscle spasms, contributing to axial pain and accelerated disc degeneration, ultimately leading to neurological compromise. Scheer et al. further emphasized that reduced cervical lordosis (CL), particularly at the fused segmental level (FSL), exacerbates spinal cord compression through anterior pathological encroachment and increased longitudinal cord tension due to tethering by dentate ligaments and nerve roots, thereby worsening clinical outcomes (8). For patients with cervical spondylotic myelopathy (CSM) complicated by kyphosis, surgical correction of sagittal alignment via anterior cervical discectomy and fusion (ACDF) is clinically imperative. In the present study, all patients underwent ACDF, either by stand-alone cages or plate cage system, were associated with a significantly increase of CL and FSL postoperatively, and the changes between two groups were comparable.

Although both fixation methods achieved immediate postoperative restoration of cervical lordosis, extensive studies suggest that PC offers superior long-term maintenance of cervical alignment. In 2019, Tao et al. (3) compared 116 patients undergoing ACDF with SA or PC. At two-year follow-up, both groups demonstrated comparable clinical improvements; however, the SA group exhibited significantly reduced cervical lordosis, fusion segmental lordosis, and intervertebral height compared to the PC group at final follow-up. Recently, Xiao et al. (35) investigated the impact of SA vs. PC on sagittal alignment in three-level ACDF. The SA group showed significantly greater loss of cervical lordosis (C2–C7 Cobb angle) at one-year follow-up compared to the PC group. Previous studies by the team also emphasize the importance of prioritizing sagittal alignment restoration when using SA for multilevel ACDF. Elias et al. (25) conducted a meta-analysis of 41 articles comparing SA and PC, concluding that PC demonstrated superior performance in maintaining disc height, cervical lordosis, and lower cage subsidence rates. Consistent with these findings, our study revealed significantly lower CL, FSL, and fused segment disc wedge in the SA group at two-year follow-up compared to the PC group, indicating more pronounced loss of sagittal alignment over time.

Cage subsidence, defined as ≥3 mm loss of the anterior height of the fused segment between immediate postoperative and final follow-up radiographs (18, 19), is influenced by multiple factors. Noordhoek et al. (18) systematically reviewed subsidence rates and found that cage-screw combinations exhibited significantly less subsidence compared to PEEK, titanium, or PMMA cages (15.1% vs. 23.5% vs. 24.9% vs. 30.2%; *p* < 0.001). However, most studies reported no significant correlation between subsidence and clinical outcomes. Opsenak et al. (36) analyzed 61 patients undergoing 1–2 level ACDF with Zero-Profile cages and identified osteoporosis as a risk factor for subsidence. Similarly, Mohamed et al. (37) demonstrated that lower bone density, as measured by the Cervical Vertebral Bone Quality Score (C-VBQ), correlated with higher subsidence rates. In our study, osteoporosis patients exhibited significantly lower FSL and fused segment disc wedge at final follow-up. Villavicencio et al. (38) and Guérin et al. (39) noted that fusion segmental alignment, rather than overall cervical lordosis, significantly influenced clinical improvement. This may explain the higher VAS-neck scores in the SA group at final follow-up, though the difference did not reach MCID, suggesting the need for larger-scale studies.

At the final follow-up, the PC group demonstrated significantly greater cervical lordosis (CL), fusion segmental lordosis (FSL), and fused segment disc wedge compared to the SA group (*p* < 0.05 for all parameters). These findings suggest that the PC system may offer superior sagittal alignment restoration, likely attributable to its pre-contoured design, which facilitates anatomical curvature reconstruction. Additionally, the locking screw mechanism enhances reduction efficacy by stabilizing intervertebral disc height and reducing cage subsidence, thereby preserving corrective outcomes over time.

Building upon prior studies that classify cervical kyphosis as reducible or non-reducible based on global cervical alignment in extension radiographs (11), our study introduced a novel classification system focused specifically on the fused segment. In this framework, reducible kyphosis was defined as the ability to restore segmental lordosis at the fused levels on preoperative extension imaging, whereas non-reducible kyphosis denoted persistent kyphotic deformity despite dynamic evaluation. Subgroup analysis revealed distinct outcomes: for reducible kyphosis, both SA and PC achieved comparable maintenance of cervical lordosis at final follow-up (*p* > 0.05). In contrast, SA demonstrated significantly greater loss of cervical and segmental lordosis compared to PC in the non-reducible subgroup (*p* < 0.05). This divergence may stem from biomechanical differences during surgical correction. Reducible kyphosis allows natural disc space opening under neck extension during anesthesia, minimizing the need for forceful intraoperative distraction. In non-reducible cases, aggressive intervertebral distraction is often required to restore disc height, increasing the risk of endplate compromise and cage subsidence (36). Excessive mechanical stress on structurally compromised endplates in non-reducible deformities could accelerate subsidence, ultimately leading to sagittal malalignment. These findings underscore the importance of preoperative dynamic imaging to assess reducibility at the fused segment, offering surgeons a practical criterion for selecting SA or PC.

Notably, although statistically significant differences in cervical and segmental lordosis were observed between the two constructs at final follow-up, the absolute magnitude of these differences was modest and did not translate into short-term functional superiority. Nonetheless, even small variations in sagittal alignment may carry biomechanical relevance for alignment preservation and long-term stability, particularly in patients with rigid or non-reducible deformities

### Limitations

This study has several limitations. First, the single-center retrospective design and relatively small sample size (*n* = 130) may restrict the statistical power and generalizability of the findings. Second, the mid-term follow-up period (median 28.5 months) may be insufficient to fully capture long-term complications, such as ASD or progressive alignment loss. Third, the absence of whole-spine radiographs limited analysis of global sagittal alignment parameters (e.g., thoracic kyphosis, lumbar lordosis), potentially confounding cervical alignment interpretation. Fourth, fusion rate was assessed via dynamic radiographs rather than CT scans—the diagnostic gold standard—which may overestimate fusion success. Fifth, bone mineral density (BMD) was evaluated using DXA based on the lowest T-score of the lumbar spine, femoral neck, and proximal femur, whereas cervical vertebral Hounsfield units (HU) measured by CT scans—a more reliable predictor of cage subsidence—were not analyzed (40). Sixth, although subgroup analyses were performed, unmeasured confounding factors may still influence outcomes, and additional stratification based on the magnitude of kyphosis may provide further insights and should be considered in future studies. Finally, the retrospective design inherently introduces potential selection bias, as construct selection was influenced by surgeon preference and implant availability. This may limit causal inference and affect the generalizability of the findings. A prospective multicenter trial with extended follow-up and advanced imaging protocols (e.g., CT-based BMD, whole-spine radiographs) is warranted to validate these findings.

## Conclusion

Both SA and PC improved neurological function and cervical lordosis in CSM patients with kyphosis. While SA reduced operative time and dysphagia incidence, PC demonstrated superior mid-term maintenance of sagittal alignment. Based on these findings, plate-cage fixation may be preferentially recommended for patients with non-reducible cervical kyphosis, whereas either construct appears to be a reasonable option in reducible deformities.

## Data Availability

The raw data supporting the conclusions of this article will be made available by the authors, without undue reservation.
